# Movement-Based Control for Upper-Limb Prosthetics: Is the Regression Technique the Key to a Robust and Accurate Control?

**DOI:** 10.3389/fnbot.2018.00041

**Published:** 2018-07-26

**Authors:** Mathilde Legrand, Manelle Merad, Etienne de Montalivet, Agnès Roby-Brami, Nathanaël Jarrassé

**Affiliations:** Sorbonne Université, CNRS, INSERM, Institut des Systèmes Intelligents et de Robotique, International Society for Intelligence Research (ISIR), Paris, France

**Keywords:** upper-limb prosthetics, movement-based control, shoulder-elbow coordinations, regression algorithms, motor strategy

## Abstract

Due to the limitations of myoelectric control (such as dependence on muscular fatigue and on electrodes shift, difficulty in decoding complex patterns or in dealing with simultaneous movements), there is a renewal of interest in the movement-based control approaches for prosthetics. The latter use residual limb movements rather than muscular activity as command inputs, in order to develop more natural and intuitive control techniques. Among those, several research works rely on the interjoint coordinations that naturally exist in human upper limb movements. These relationships are modeled to control the distal joints (e.g., elbow) based on the motions of proximal ones (e.g., shoulder). The regression techniques, used to model the coordinations, are various [Artificial Neural Networks, Principal Components Analysis (PCA), etc.] and yet, analysis of their performance and impact on the prosthesis control is missing in the literature. Is there one technique really more efficient than the others to model interjoint coordinations? To answer this question, we conducted an experimental campaign to compare the performance of three common regression techniques in the control of the elbow joint on a transhumeral prosthesis. Ten non-disabled subjects performed a reaching task, while wearing an elbow prosthesis which was driven by several interjoint coordination models obtained through different regression techniques. The models of the shoulder-elbow kinematic relationship were built from the recordings of fifteen different non-disabled subjects that performed a similar reaching task with their healthy arm. Among Radial Basis Function Networks (RBFN), Locally Weighted Regression (LWR), and PCA, RBFN was found to be the most robust, based on the analysis of several criteria including the quality of generated movements but also the compensatory strategies exhibited by users. Yet, RBFN does not significantly outperform LWR and PCA. The regression technique seems not to be the most significant factor for improvement of interjoint coordinations-based control. By characterizing the impact of the modeling techniques through closed-loop experiments with human users instead of purely offline simulations, this work could also help in improving movement-based control approaches and in bringing them closer to a real use by patients.

## 1. Introduction

Advances in mechatronics and robotics over the last years have led to the production of more biomimetic active prostheses with more and more degrees of freedom (DoFs). Upper limb amputees can thus be proposed complex active mechatronic devices like polydigital hands or whole arm prostheses like the Luke Arm by Deka (Resnik et al., 2013) or the modular arm by the Applied Physics Laboratory of Johns Hopkins (Johannes et al., 2011), among other examples. However, while the hardware improved, there remains a lack of natural, easy and intuitive control of these artificial limbs with numerous active DoFs (Engdahl et al., 2012; Cordella et al., 2016). Conventional myoelectric control commands these multiple DoFs with only one or two muscles, which leads to complex and sequential control schemes. Indeed, depending on the amputation level, there can be the hand, the wrist and the elbow to control at the same time, each with at least two distinct actions to pilot. To improve myoelectric control in such a case, solutions like pattern recognition have been developed for more than 20 years (Saridis and Gootee, 1982; Park and Lee, 1998; Chu et al., 2006). Myoelectric control via time-invariant muscle synergies is also explored to allow continuous and simultaneous control of multiple DOFs (Lunardini et al., 2016). Yet, with all the limitations of the EMG signals measurement and its decoding [electrode shift, sensibility to perturbations like sweat or skin impedance, etc. (Castellini et al., 2014), leading to a robustness issue], there is a renewal of interest in movements, that humans are more likely to control than individual muscle contractions (see works by Kaliki et al., 2008, 2013; Popovic and Popovic, 2001, 2002; Alshammary and Bennett, 2016 for instance). It is actually easier to master a sequence of movements than a sequence of contractions/co-contractions, which is highly unnatural. We indeed receive numerous sensory feedbacks of our own movements (vision but also proprioception or tactile), compared to the one of our muscular activity. Movement-based control approaches aim to create a more intuitive and natural control by using the motion of the residual limb to predict the movement of the prosthesis. Indeed, it has been showed that one way the Central Nervous System (CNS) deals with the redundancy of the human body is to control synchronously several muscles or joints, by grouping them into “synergies” (which exist at the muscle and at the joint levels). For example, for a given space and task type, there exist some synergies synchronizing shoulder and elbow movements (Soechting and Lacquaniti, 1981; Lacquaniti and Soechting, 1982; Lacquaniti et al., 1982; Cirstea et al., 2003). These synergies can be modeled to then determine elbow motions from shoulder motions (Popovic and Popovic, 2001; Kaliki et al., 2008; Farokhzadi et al., 2016). Exploiting synergies could especially be useful in prosthetics control since regression methods could be used to predict motion of a distal prosthetic joint from motion of residual proximal joints.

Of course, it is important to remind that different tasks and motion spaces are associated to different synergies. It seems therefore difficult to use movement-based control to predict every motion, as each of them requires a different model; some voluntary control would always be needed. Nonetheless, for some given generic movements from the Activities of Daily Living, there could be a functional gain for patient if, for fast motion like reaching, part of the prosthesis joints was synchronously and automatically controlled, avoiding a fatiguing and slow sequential decomposition of joint actions. In this work, we thus focused on reaching tasks, for which people do not naturally concentrate on the intermediate joint control, making this motion perfectly adapted to movement-based control. For now, our approach is hybrid: movement-based control does not totally replace myoelectric control but substitutes it only for the elbow even if synergy-based control could be used for the wrist (Montagnani et al., 2015). Long-term goal would be to control both elbow and wrist with joint synergies; we chose to focus first on the elbow. Joint synergies yet cannot control the hand as it is not part of a synergistic scheme with more proximal joints.

Some studies have already been conducted on movement-based control for elbow-shoulder motion. Merad et al. (2016a,b), for instance, used Radial Basis Function Networks (RBFN), one of the simplest Artificial Neural Networks (ANNs), to estimate flexion/extension elbow angular velocity from shoulder Euler angular velocities, measured with embedded Inertial Measurement Units. In a wider context, Kaliki et al. (2013) developed an inferential control scheme to command elbow flexion/extension, forearm pronation/supination and opening/closing of the hand at the same time. They combined three ANNs and proportional control that took shoulder rotational and/or translational movements as inputs (recorded with a magnetic tracking system) and predicted the outputs cited above. In addition to the work of Kaliki et al. and Merad et al. several other studies on shoulder-elbow coordinations have been published (Popovic and Popovic, 2002; Iftime et al., 2005; Mijovic et al., 2008; Farokhzadi et al., 2016). At this time, two points can be raised:

- There is not one accepted regression method to model shoulder-elbow synergies, each research group or study uses a different one, without any clear justification;
- The validation of the models are generally performed offline, through simulations, or in a virtual reality environment. This limits the evaluation of the robustness of the model in real case scenario (i.e., in a closed-loop with a human user adapting in return to the prosthetic reaction).

This study addresses these two issues. Starting from the fact that none of the cited studies has used a linear regression technique, we first wondered whether it was really unsuitable (whereas it has been showed that shoulder-elbow synergies can be approximated by a linear relationship; Micera et al., 2005). Then, we wanted to compare the prediction ability of several models to objectively and reliably determine the best modeling tool for the control of a prosthetic elbow. We here focused on three relatively simple methods: RBFN, the simplest ANN, which was shown to correctly model shoulder-elbow synergies (Iftime et al., 2005); Principal Components Analysis (PCA), to test the prediction ability of a linear regression technique; and Locally Weighted Regression (LWR), whose complexity is between PCA and RBFN. We conducted a preliminary experimental session, with fifteen healthy subjects that performed reaching movements, to gather training data and build the three generic interjoint coordination models. Once the models were implemented in the prosthesis, a second experimental session was conducted with ten other healthy subjects who performed the same tasks as in the preliminary session but with the prosthesis substituting to their natural arm. The prosthesis was controlled through the mobilization of the subjects' shoulder as the control input. To determine the best regression methods for prosthetic control, six metrics, that characterize the task achievement, the joint motions and the body compensations, were assessed.

In this paper, we thus focus on the elbow-shoulder synergies to automatize a prosthetic elbow during reaching tasks. Real tests, in “closed loop” situation, were conducted to compare the three elbow-shoulder coordination models obtained with RBFN, PCA, and LWR respectively. During these tests, the participants could directly react to the system behavior, which is closer to real life scenario and gives more weight to the reflection on the models robustness than fixed offline data simulation.

## 2. Materials and methods

### 2.1. Preliminary session: training data acquisitions

**Figure 1:**
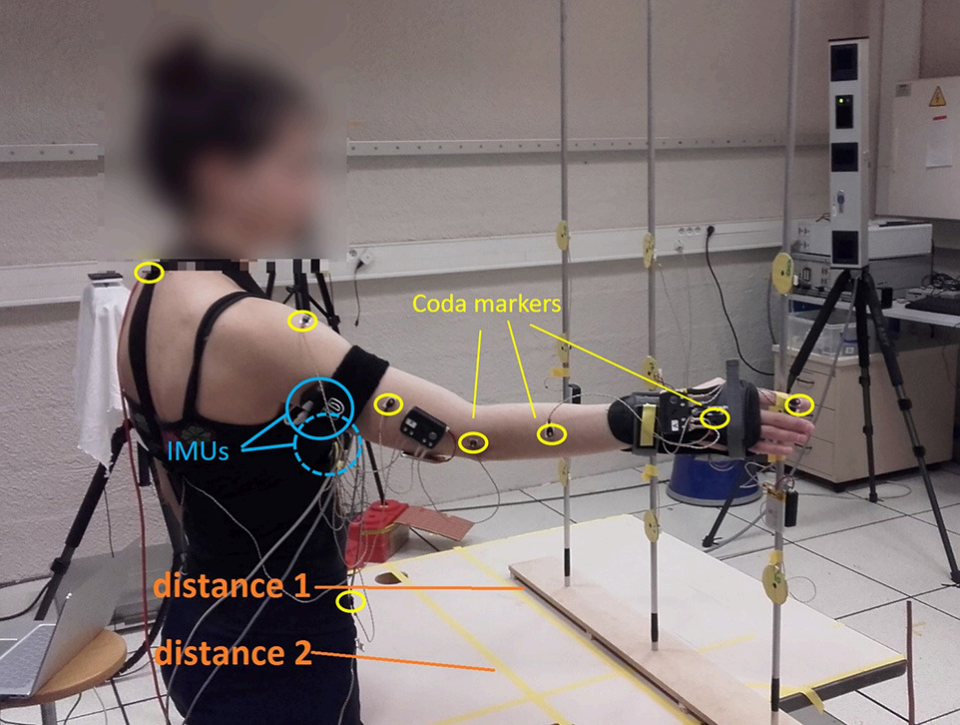
Experimental set-up for training data recordings: participants performed natural reaching movements toward 18 targets (9*2 distances). x-IMUs are placed over the arm and the trunk; Coda markers on the arm, shoulder, and trunk. Written informed consent for publication of images was obtained from the participants.

**Figure 2:**
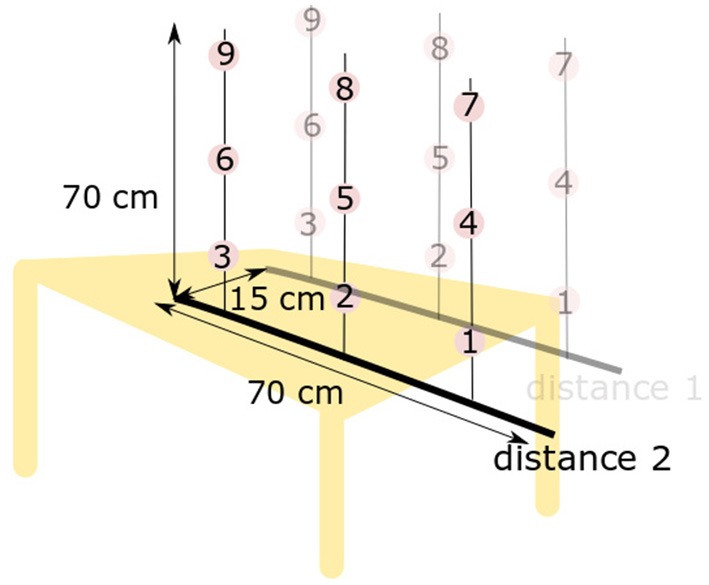
Localization of targets to reach.

**Figure 3:**
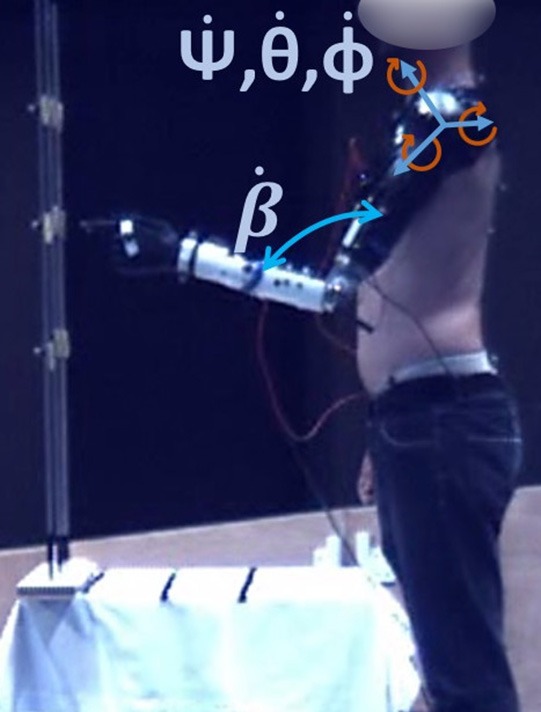
Inputs and output of the models. The inputs are $\overset{\cdot }{\psi },\overset{\cdot }{\theta },\overset{\cdot }{\phi }$, the ZYX Euler angular velocities of the shoulder, in the trunk frame. The output is $\overset{\cdot }{\beta }$, the elbow flexion/extension angular velocity. Written informed consent for publication of images was obtained from the participants.

To build and train the coordination models, data of motions from healthy subjects are required. These training data were collected from fifteen healthy subjects (different from those who participated to the second session) who performed pointing movements with their natural arm. Kinematics was recorded with motion capture (Figure 1). Ten subjects used their right arm, ten their left arm (five subjects participated twice). This work was carried out in accordance with the recommendations of the Université Paris Descartes ethic committee CERES. Subjects provided written informed consent to participate in the study, in accordance with the Declaration of Helsinki. Two Inertial Measurement Units (x-IMUs from x-io technologies©), a Codamotion (a camera-based motion capture system from Charnwoods Dynamics, Leicestershire, UK) and a Nintendo Wii^TM^ balance board were used to record the movements. IMUs, one located on the latero-posterior part of the arm, the other on the trunk, at the sternum level, recorded the arm orientation in the trunk coordinate system, represented by quaternion values and then transformed into ZYX Euler angles. Codamotion markers were placed on the hand, forearm, arm, shoulders and hips to record elbow flexion/extension angle as well as other kinematic parameters for further analysis. The balance board was used to measure the variations of the weight repartition at the feet level when performing the task. Subjects had to reach nine targets at two different distances (18 targets in total), whose height and position were adapted to subjects' morphology (the length of the subject's arm minus 10 cm defined the first distance, the second one was 15 cm closer. Targets 1, 2, and 3 were at the hip level, targets 7, 8, and 9 were at the shoulder level, targets 4, 5, and 6 were in-between see Figure 2). Each target was reached three times with pause between each movement. No specific instruction were given to the participants, to let them move naturally. Only the initial position was imposed: subjects were asked to start with the humerus along the body and the elbow flexed at 90°. Shoulder ZYX Euler angular velocities, computed in the trunk frame, and elbow flexion/extension angular velocity (obtained from IMUs and Codamotion markers respectively) were collected and used to train the three models offline, thanks to a Matlab (Mathworks Inc.) script. As the aim is to predict elbow motions from shoulder ones, the inputs of the models were the shoulder data (ZYX Euler angular velocities in the trunk frame) and the output was the elbow data (flexion/extension angular velocity, see Figure 3). We chose to use joint velocities to avoid any dependence on the initial position. Shoulder Euler angles were selected as input data since they are commonly used in shoulder-elbow coordination modeling (Lacquaniti et al., 1982; Popovic and Popovic, 2001; Wu et al., 2002; Kaliki et al., 2008). The kinematic data were filtered (low-pass filter with a cut-off frequency of 5 Hz) and segmented. The start and end of the movements were automatically determined with a Matlab script, using a threshold on the hand velocity profile (30% of the maximum velocity ± an offset adapted to each subject). Only the go were used for training the models.

### 2.2. Models

Let *f* be the function that approximates the relationship between the selected inputs/outputs sets. For PCA, used for regression as in Vallery and Buss (2006), we have, for a given input vector **x** (the three shoulder Euler angular velocities for one time sample in our case):

(1) $$f(x)=\Gamma_{2}\Gamma_{1}^{+}\text{x}$$

with Γ the matrix of principal components of the training data, Γ_1_ and Γ_2_ the corresponding sub-matrices. The first two Principal Components were kept since they were enough to account for 98% of the total variance. We thus have $\Gamma_{1}\in {\mathbb{R}}^{3\times 2}$ and $\Gamma_{2}\in {\mathbb{R}}^{1\times 2}$. $\Gamma_{1}^{+}={\left(\Gamma_{1}^{T}\Gamma_{1}\right)}^{-1}\Gamma_{1}^{T}$ is the left pseudo-inverse of Γ_1_.

For LWR, the output is defined as:

(2) $$f(x)=\sum_{e\;=\;1}^{E}\phi (x,\theta_{e})\;\cdot \;a_{e}{}^{T}x,$$

(with *E* the number of local linear models, ϕ the weighting functions of these models -here Gaussian functions-, θ_*e*_ which accounts for the localization and **a**_**e**_ parameters of the linear models) (Stulp and Sigaud, 2015). The number of local linear models, which minimized the residual error (between real and predicted output), was set to 2 after cross-validation.

For RBFN, we have:

(3) $$f(x)=\sum_{e\;=\;1}^{E}w_{e}\;\cdot \;\phi (x,\theta_{e}),$$

(with the radial basis functions ϕ, set as Gaussian functions, and *w*_*e*_ the weight for each function, determined with linear least square method) (Stulp and Sigaud, 2015). The number of basis functions *E*, that minimized the residual error, was set to 5 after cross-validation.

### 2.3. Experimental session: testing the models in closed loop situation

Ten different healthy subjects, who did not contribute to the collection of training data, participated in the second experimental session. They were equipped with a prosthetic elbow prototype with one active DoF (flexion/extension of the elbow). The prototype was attached laterally to an elbow orthosis worn by the subject (attached to his arm), installed such that the prosthesis rotation axis was aligned with the natural elbow flexion/extension axis of the participant. The elbow orthosis blocked any motion of the natural elbow (it was fixed at 90° during the whole experiment). Five subjects used the prototype to the right, five to the left. The control models were trained on the data of the preliminary experimental session from the right and left arm group respectively.

#### 2.3.1. Prosthetic elbow prototype

**Figure 4:**
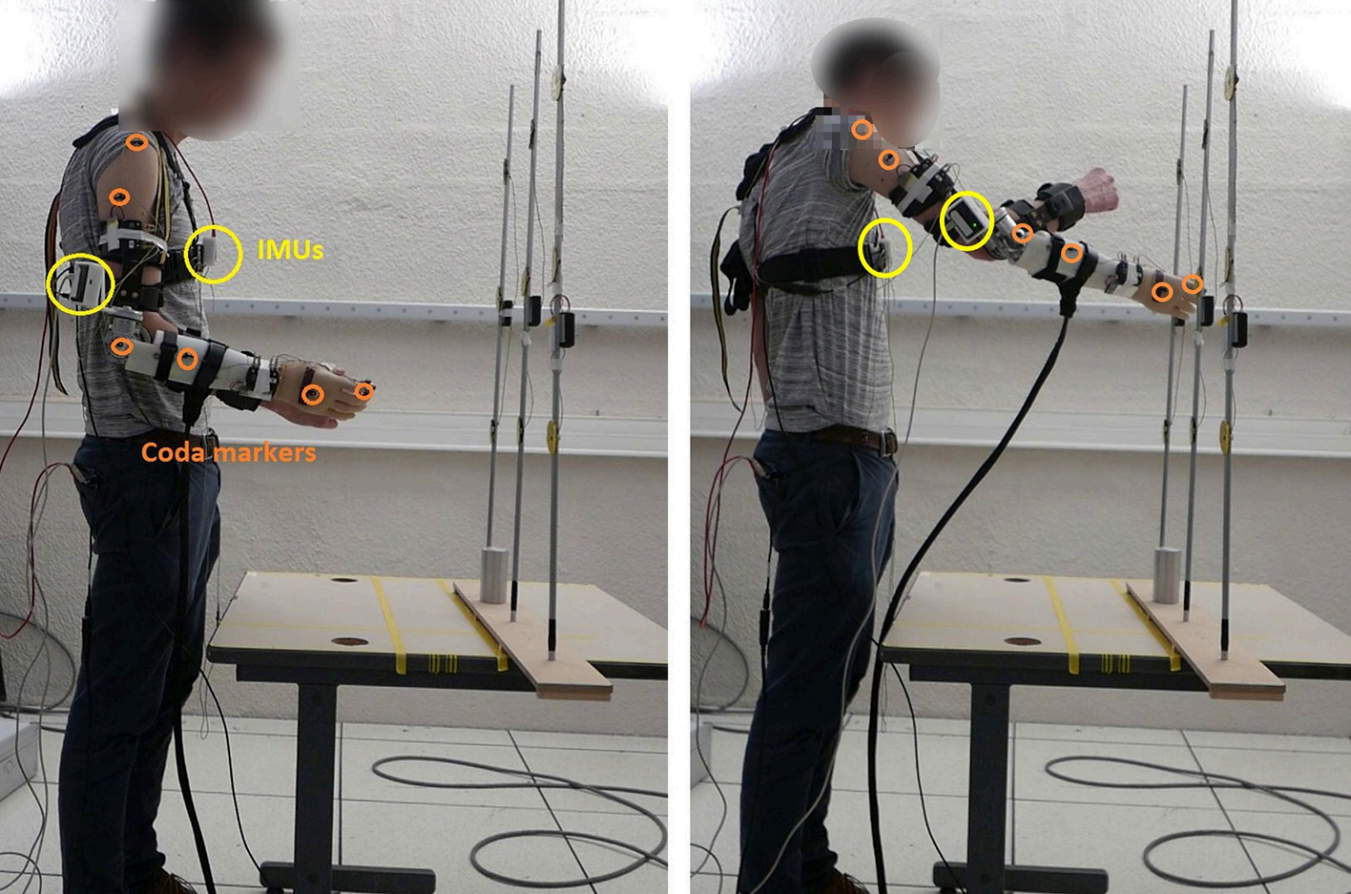
Experimental set-up: participants wore the prosthetic elbow as a supernumerary arm thanks to an orthosis. x-IMUs are put on the arm and on the trunk and Coda markers on the arm, shoulder, and trunk; a balance board is placed under participant's foot. Task consists in reaching the targets with the prosthesis. **Left**: initial position. **Right**: reaching movement. Written informed consent for publication of images was obtained from the participants.

The prosthetic elbow is a 1-DoF (flexion/extension) prototype whose functional characteristics are based on the ones of commercialized active elbow prostheses (10 N/m of nominal torque, 80°/s of nominal speed). The angular velocity is controlled by a DC motor driver (Ion motor control, Ltd) via an optical encoder placed on the motor rear shaft (resolution of 2,048 ppr and gear ratio of 1:1,000). The prototype is controlled by a Raspberry PI, which controls the DC motor driver. It reads data from two x-IMUs (Xio Technologies, Ltd.) placed on the subjects arm and trunk, at the same location as for the preliminary experimental session. The IMUs gave quaternion values representing the arm orientation, from which ZYX Euler angular velocities of the shoulder, in the trunk frame ($\overset{\cdot }{\psi },\overset{\cdot }{\theta },\overset{\cdot }{\phi }$) were computed. IMUs were reset at the beginning of each experimental session, and their position remains unchanged during the whole experiment. They are the only devices used for control. The Codamotion and balance board were used for analysis purpose only; specific Coda markers were placed on the arm, the shoulders and the hip (see Figure 4). A prosthetic hand, blocked in an open posture (forming a u-shape in the horizontal plane), was placed at the extremity of the prosthetic limb. The subjects reached the targets by placing this hand around them.

#### 2.3.2. Experimental set-up

Participants were asked to use the prosthesis to reach the same eighteen targets as for the preliminary experimental session. We did not ask them to reach new targets because this study particularly focused on the robustness of the interjoint coordination models (obtained through RBFN, PCA, and LWR) to the inter-subject variability. We were interested in the prosthesis response to different motor behaviors and kinematics. Elbow angular velocity $\overset{\cdot }{\beta }$ was estimated by the different regression models from $\overset{\cdot }{\psi },\overset{\cdot }{\theta },\overset{\cdot }{\phi }$, the shoulder Euler angular velocities, computed in the trunk coordinate system, obtained from the IMUs. The initial position, to which the participants had to come back after every movement, was defined with the prosthetic elbow at 90 degrees and subject's humerus at zero degrees, along the body. The task was limited to the go (from initial position to target), the return of the prosthesis (from target to initial position) was automatic. The end of the movement was defined by the end of the prosthesis motion toward the target (elbow velocity set to zero when the shoulder angular velocities dropped below a chosen threshold). Subjects were asked not to correct the final reached position with visual feedback, even if the prediction was bad. Each target was reached 3 times, each time with a different model. The order of models used for control was randomized before the experimental session, and subjects were not aware of this order. Models were implemented in the Raspberry PI which controls the prosthesis. $\overset{\cdot }{\psi },\overset{\cdot }{\theta },\overset{\cdot }{\phi }$, obtained from xIMUs, were sent as model inputs. The total experimental session (placement of the markers and the prosthesis, reaching tasks and removal of the markers and the prosthesis) lasted approximatively 2 h.

#### 2.3.3. Performance quantification

**Figure 5:**
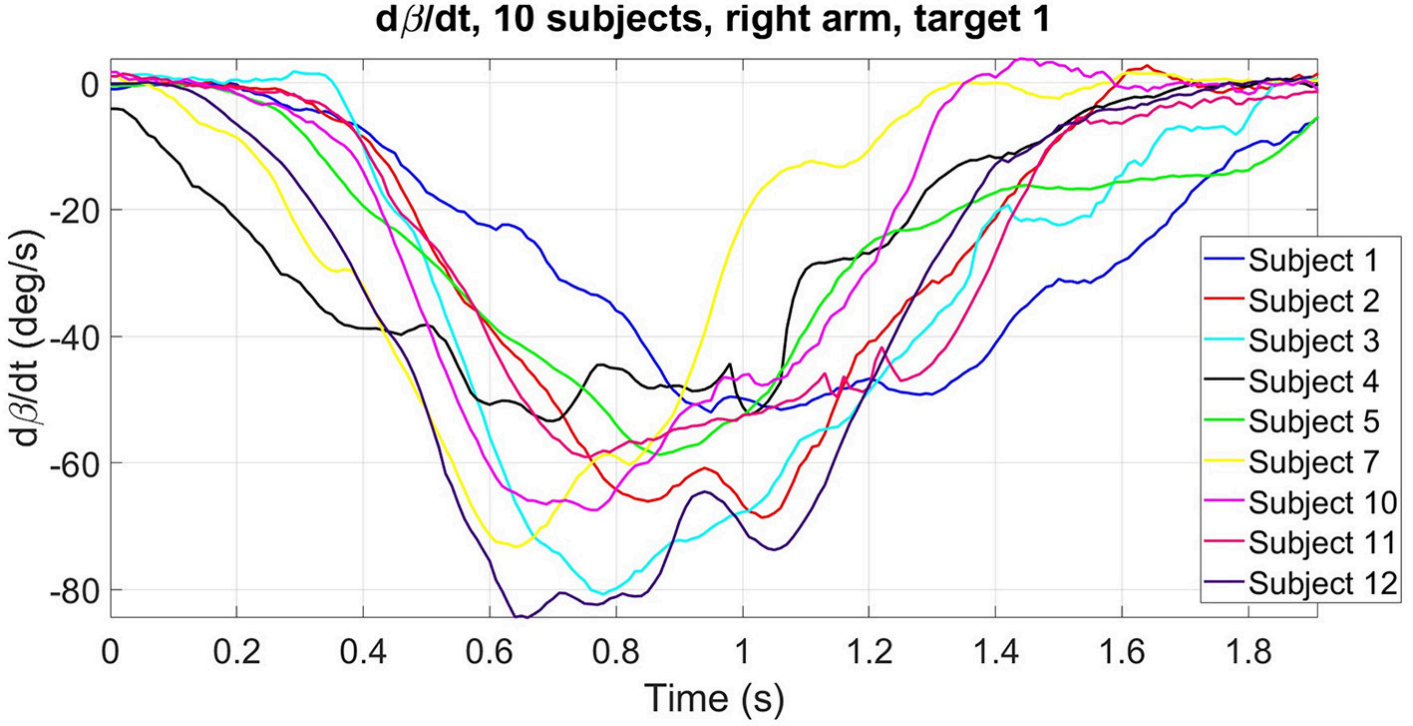
Illustration of inter-subject variability in elbow flexion/extension angular velocity. Example of time evolution of $\overset{\cdot }{\beta }$ (target 1) for the ten healthy subjects that performed the preliminary session with their right arm.

**Figure 6:**
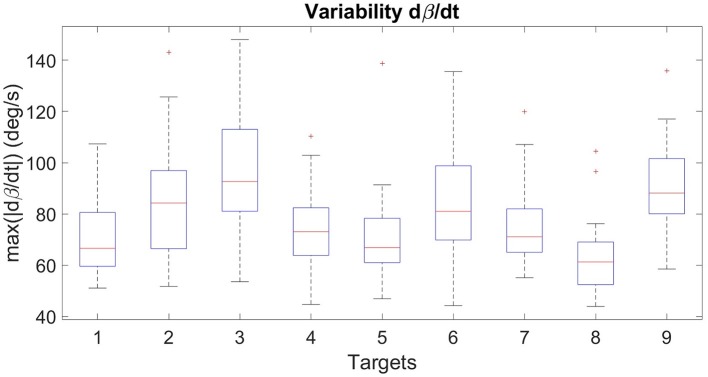
Box plot of the maximum of $\left|\overset{\cdot }{\beta }\right|$ for the ten healthy subjects that performed the preliminary session with their right arm, for the nine targets of distance 1.

Evaluating whether a movement was correctly performed is a complex task. Indeed, despite some characteristics shared among subjects in reaching motions, there is a significant inter-subject variability that prevents the use of traditional error values. Figure 5 illustrates the inter-subject variability of $\overset{\cdot }{\beta }$ for the ten subjects that performed reaching motions with their right arm in the preliminary experimental session (without the prosthesis). On the box-plot of the maximum of $\left|\overset{\cdot }{\beta }\right|$ (Figure 6), we can see that the range of variation is large and that there is even some outliers identified, whereas all the motions were correct. Considering an average healthy $\overset{\cdot }{\beta }$ and compute an error with respect to it for a given motion is thus not relevant. Moreover, the targets can be correctly reached but with the help of compensatory movements (such as trunk flexion or rotation) that have to be avoided. Musculoskeletal pain and overuse injuries are actually a well-known problem for the upper-limb amputee population (Kontson et al., 2017; Postema, 2017). Error value of $\overset{\cdot }{\beta }$ only concentrates on functional performance and does not take this point into account. For these reasons, we developped sixteen features to evaluate the performance of the models used for prosthetic control. They were defined in order to give a measure of the achievement of the task, the natural (or unnatural) aspects of the arm movements and the importance of the body compensations. Six of the most relevant metrics are presented here, since the others lead to the same conclusion (see **Appendix** for the exhaustive list) :

- The distance between the final position of the index finger and the target to reach, δ. As the subjects were asked not to correct the final position of the prosthesis, δ gives a measure of the good (or bad) achievement of the task;
- The delay Δ_*t*_ introduced by the control schemes before the activation of the elbow. It is defined by the difference between the beginning of the shoulder motion (when the first Euler angular velocity is higher than 5% of its extremum) and the beginning of the elbow motion ($\overset{\cdot }{\beta }$ higher than 5% of its extremum). It illustrates how fast the model reacts to the subject's command;
- The curvature of the trajectory, *c*, that illustrates the deviation of the hand from a straight line trajectory toward the target. It is defined as
  (4) $$\begin{array}{r} c=\frac{\text{max}\left(\left||\vec{P\left(t\right)H\left(t\right)}|\right|\right)}{\left||\vec{P\left(t_{final}\right)P\left(t_{0}\right)}|\right|} \end{array}$$
  with *P* the end-effector position at each time step and *H* the orthogonal projection of *P* on the straight line (*P*(*t*_0_)*P*(*t*_*final*_)). It measures the natural aspect of the movement.;
- The smoothness *s* of the elbow angular velocity ($\overset{\cdot }{\beta }$) measured by its spectral arc length (Balasubramanian et al., 2012). During the experiment, we observed that, for some models, the extension of the elbow (and so the arm movement) was jerky, which was very unpleasant for the user. It is thus important to quantify the smoothness of the movement to select a model that predicts a natural (i.e., smooth and fluid) motion;
- The final angular posture of the elbow, final β;
- The amplitude ratio of the force on the ipsilateral feet, *a*,
  (5) $$\begin{array}{r} \frac{F_{ips}^{t_{f}}-F_{ips}^{t_{0}}}{F_{ips}^{mean}+F_{contra}^{mean}} \end{array}$$
  (with $F_{ips}^{t_{f}}$ and $F_{ips}^{t_{0}}$ the force on the ipsilateral feet at the end and the beginning of the movement respectively, and $F_{ips}^{mean}$ and $F_{contra}^{mean}$, the mean of the force on the ipsilateral and contralateral feet, respectively). It is given in percentage of total force applied on both foot. It measures how much the subject moves its center of mass and thus moves its trunk laterally from the start to the end of the reaching. It is a direct measure of the body compensations.

Values of these metrics for prosthetic motions were compared with values for motions performed without the prosthesis (motions performed during the preliminary session, later called “natural” motions), except for δ that is zero for natural motions (the target was always perfectly reached without the prosthesis).

## 3. Comparison of the models

### 3.1. Results

**Figure 7:**
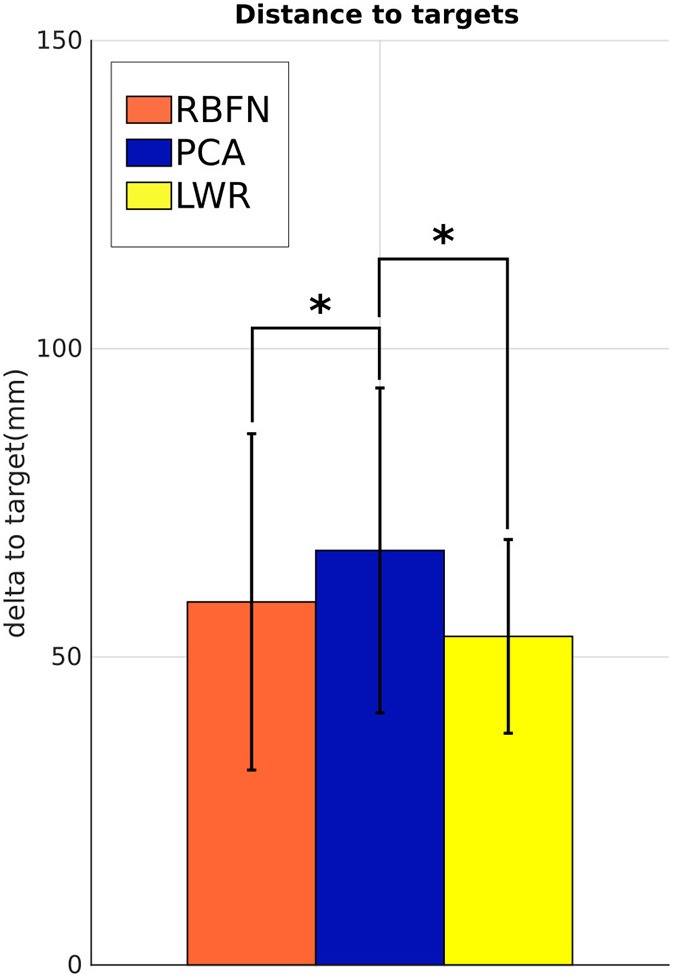
Distance δ between the end-effector of the prosthetic hand and the target to reach. Values are averaged over subjects and targets. There is no value for natural reaching motions without prosthesis as the task was always perfectly achieved in the preliminary session. *indicates a statistically significant difference (*p* < 0.05).

δ and Δ_*t*_ were averaged over subjects and targets to have one global error value per model. The curvature, *c*, and the spectral arc length of $\overset{\cdot }{\beta }$, *s*, were first averaged over subjects, to have one value per model and targets, and then over targets to simplify the analysis. The final extension of the elbow, β_*final*_, and the amplitude ratio of the ipsilateral force, *a*, were only averaged over subjects (the average over targets does not make any sense since the two metrics directly depend on target location). Statistical analysis (Wilcoxon test for difference between models and ANOVA of Friedman for targets location dependency), performed on Statistica®, was conducted for every metrics except β_*final*_ because of the lack of data for some targets. The final position error, δ (see Figure 7), is bigger for motions induced by PCA controller than for motions induced by RBFN or LWR controllers (+10 and +15 mm respectively, *p* < 0.05). δ of motions controlled by LWR is the smallest (53 mm) and its standard deviation is smaller than the one of δ of RBFN- or PCA-controlled movements.

**Figure 8:**
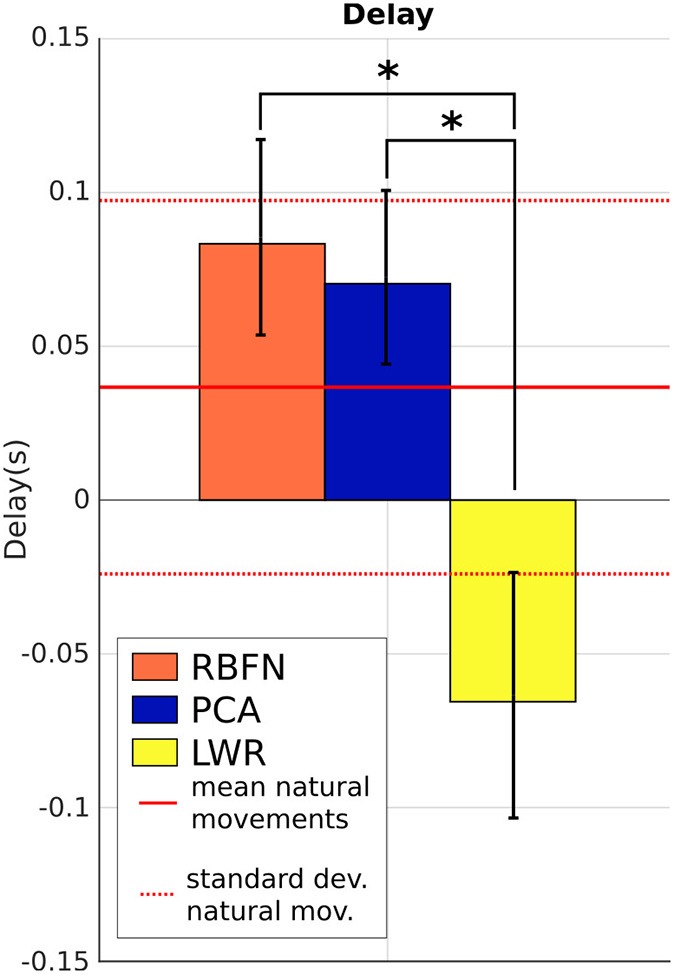
Δ_*t*_, delay between the shoulder and the elbow motions, that illustrates the time of response of the model, mean over subjects and targets. Straight and dotted red lines are respectively the mean and standard deviation of the values from natural reaching motions without the prosthesis. *indicates a statistically significant difference (*p* < 0.05).

On Figure 8, we note that there is a natural delay between shoulder and elbow motions, which is most of the time positive (the elbow moves after the shoulder). The sign of Δ_*t*_ has no evident correlation neither with the target location nor with the subjects. We can still see that, compared to the natural Δ_*t*_, the most reactive model is PCA, with 7 ms of delay. RBFN is a bit slower, with 8 ms. Both stay in the natural baseline. LWR shows a different behavior since , on average, the elbow starts moving before the shoulder (Δ_*t*_ is -60ms). Very small shoulder angular velocities are enough to cause elbow motion. Δ_*t*_ of LWR is thus significantly different from the one of RBFN and PCA but also from natural Δ_*t*_ (*p* < 0.05).

**Figure 9:**
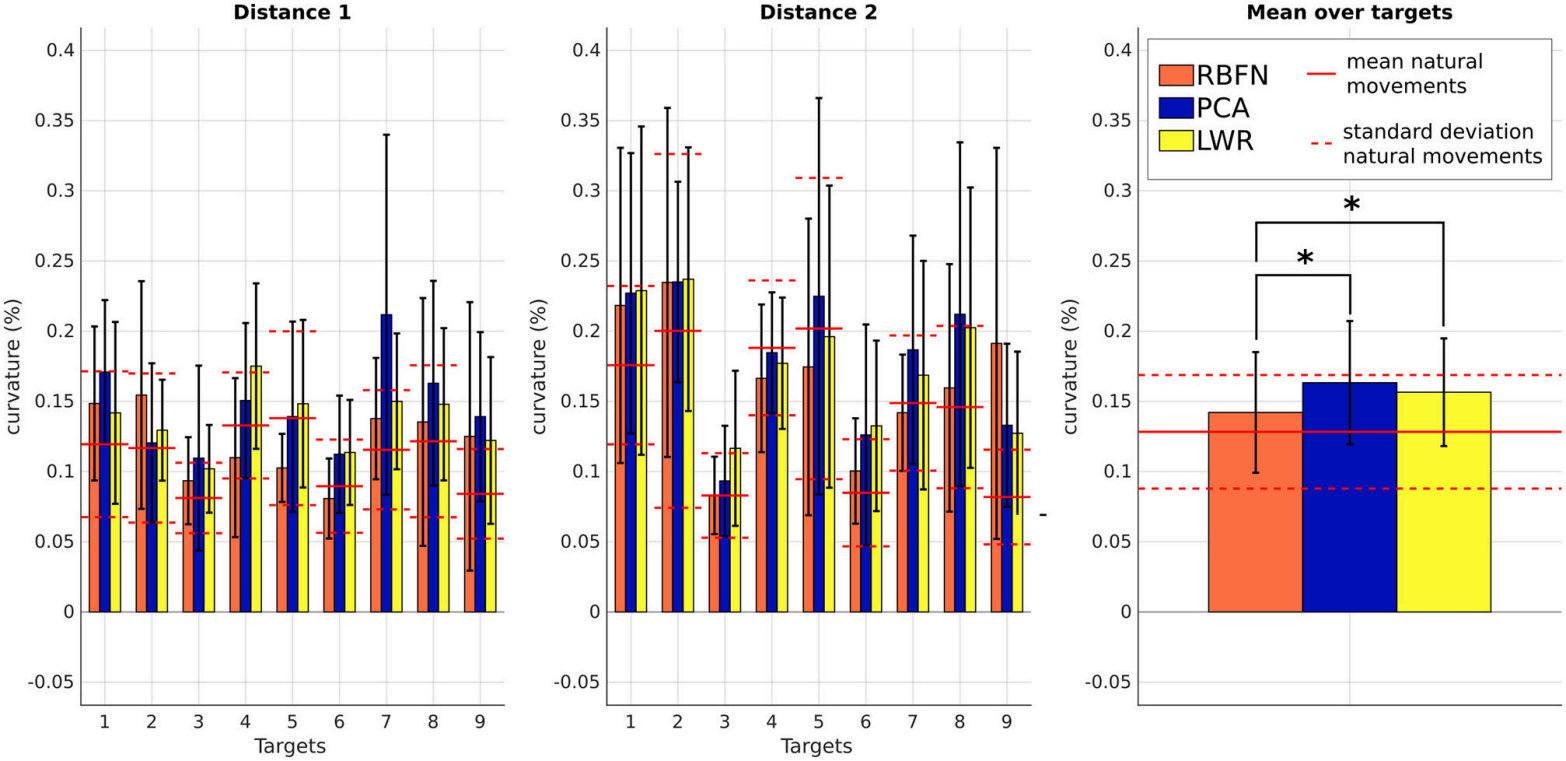
Curvature *c* of the movements obtained by the three regression models. Values are averaged over subjects. From left to right: *c* for targets of distance 1, *c* for targets of distance 2 and mean of *c* over targets. Straight and dotted red lines are respectively the mean and standard deviation of the values from natural reaching motions without the prosthesis (preliminary experimental session). Numbers correspond to the targets location, see Figure 2. *indicates a statistically significant difference (*p* < 0.05).

On Figure 9, we first see that *c* depends on the target reached (p < 0.05). It is an expected result as the curve described by the end-effector varies according to the height and the lateral position of the targets. For most of the targets, movements estimated by PCA and LWR controllers have a larger curvature than those estimated by RBFN controller or than natural motions. This is confirmed by the mean of *c*, whose values for PCA and LWR are significantly different from the value of RBFN-controlled motions (*p* < 0.05) or from the one of natural motions (*p* < 0.05). Reaching motions performed with PCA and LWR control have thus a less natural trajectory than those performed with RBFN control, even though they still stay in the range of natural motions.

**Figure 10:**
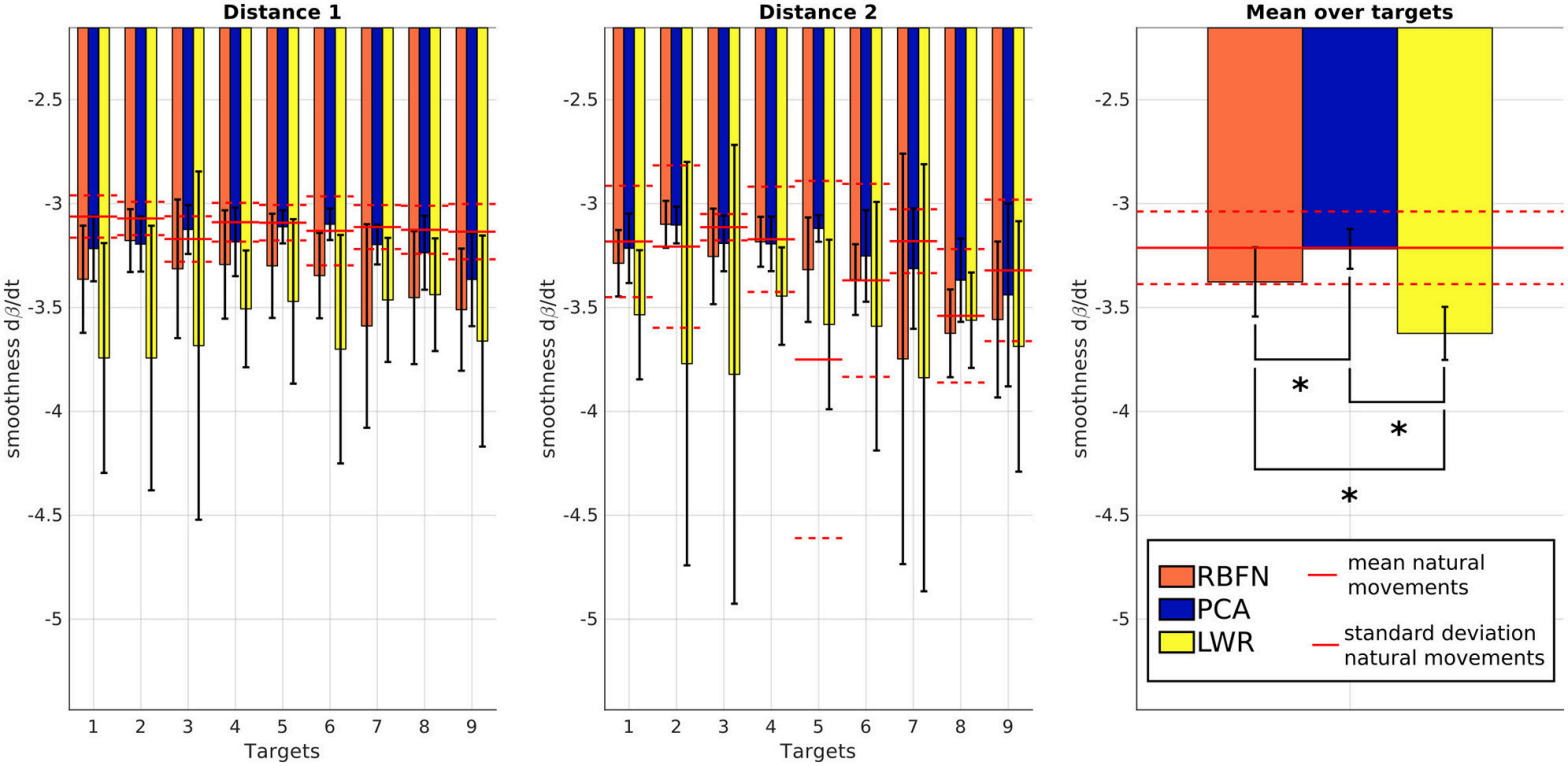
*s*, spectral arc length of $\overset{\cdot }{\beta }$, for the three regression models. The more negative, the less smooth. Values are averaged over subjects. From left to right: *s* for targets of distance 1, *s* for targets of distance 2 and mean of *s* over targets. Straight and dotted red lines are respectively the mean and standard deviation of the values from natural reaching motions without the prosthesis. Numbers correspond to the targets location, see Figure 2. *indicates a statistically significant difference (*p* < 0.05).

**Figure 11:**
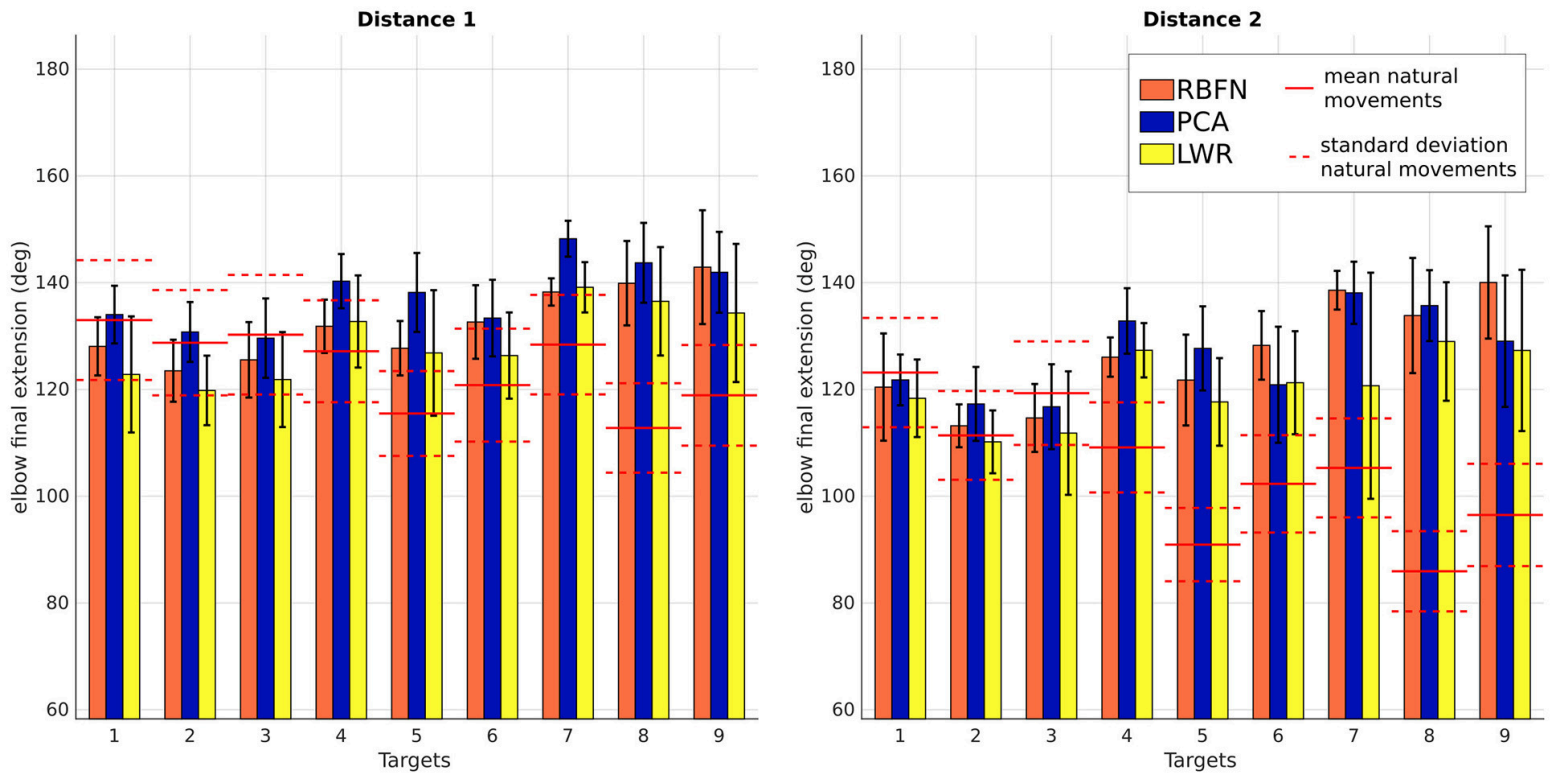
Final β, elbow final extension, of the movements obtained with the three regression models. Values are averaged over subjects. From left to right: β_*final*_ for targets of distance 1, β_*final*_ for targets of distance 2. Straight and dotted red lines are respectively the mean and standard deviation of the values from natural reaching motions without the prosthesis. Numbers correspond to the targets location, see Figure 2.

Concerning the smoothness *s*, the more negative, the less smooth is the motion. *s* does not depend on the target location for PCA-controlled, LWR-controlled and natural motions but depends on location for RBFN-controlled motions (*p* < 0.05). On Figure 10, we can quickly notice that motions made with LWR control are always less smooth than all other modes of control (RBFN, PCA and natural). *s* values of LWR are indeed significantly different from natural ones (*p* < 0.05 for 14 targets out of 18). *s* values of RBFN are significantly different for 10 targets out of 18 but are still lower than *s* values of LWR and the mean value of *s* for RBFN is in the natural baseline (i.e., lower than mean+standard deviation of smoothness for natural movements). PCA provoked significantly less smooth movements for only 3 targets out of 18 and the mean value of *s* for PCA is very close to the one of natural motions (−3.218 and −3.213, respectively). Figure 11 first shows that the elbow is too extended, in the final posture, with all regression models for the three higher targets of distance 1 and the six higher targets of distance 2. The range of β_*final*_ is smaller for motions with the prosthesis than for natural motions. The natural variations of β_*final*_ are not fully reproduced with the prosthesis, maybe because reaching of higher and/or closer targets involve slightly different joint synergies, as explained in the introduction. β_*final*_ especially discriminates PCA control since its estimation by this technique is higher than the normal extension and the one predicted by RBFN and LWR. This higher extension can explain the bigger δ of the movements with PCA control, observed Figure 7.

**Figure 12:**
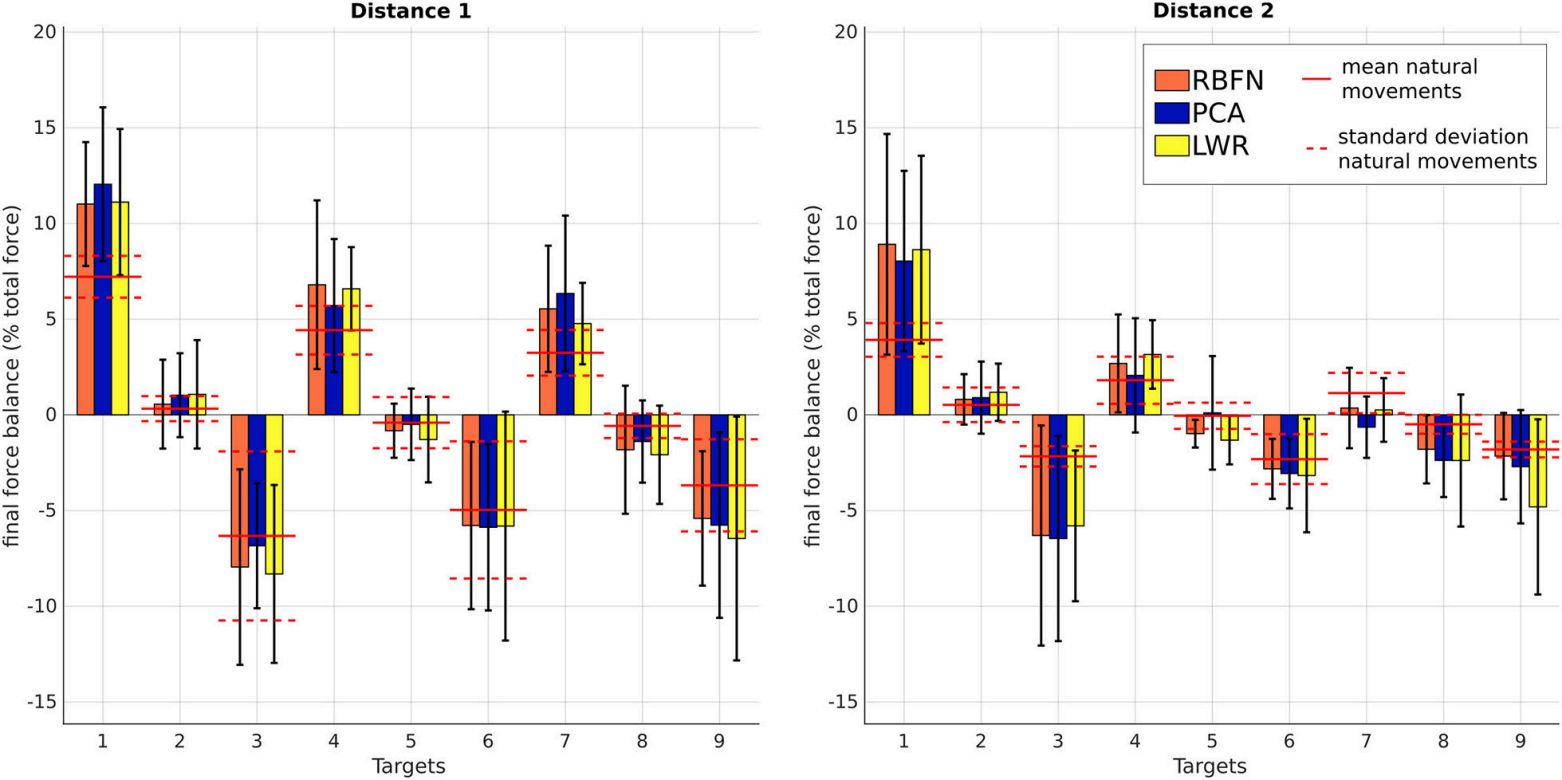
*a*, amplitude ratio of the force on the ipsilateral feet, mean over subjects. From left to right : *a* for targets of distance 1, *a* for targets of distance 2. Straight and dotted red lines are respectively the mean and standard deviation of the values from natural reaching motions. Numbers correspond to the targets location, see Figure 2.

Finally, Figure 12 shows that there are important body compensations with the prosthesis, whose amplitude depends on the target side location. These compensations may be mainly due to the weight repartition of the prosthesis which is different from the one of a natural arm, to the orthosis discomfort and/or to the shift of the prosthetic forearm relatively to the humeral axis. The body motions caused by the three regression models are significantly different from natural body motions (*p* < 0.05), but there is no significant difference between models.

### 3.2. Discussion

With these six metrics, the robustness (capacity to control the prosthesis in closed loop situations) of the models considered, the delay of their response and their generalization to new subjects can be analyzed. It can be seen that:

- Control obtained through movement estimation by PCA creates smoother movements. This seems normal as PCA is a linear model and thus has an effect similar to a low-pass filter. The timing of shoulder and elbow motions is close to the natural one, there is no annoying response delay. However, the movements created by this control have a bigger error in final position and do not have a natural trajectory. They are wider (larger curvature) and, even if $\overset{\cdot }{\beta }$ is smooth, it is overestimated (resulting in too important elbow extensions);
- Control obtained through movement estimation by LWR predicts globally correct elbow movements (except for highest targets of distance 1 and up and middle targets of distance 2, which were more difficult to reach because of their localization) and leads to the smallest final error in position. Nevertheless, the movements have a larger curvature, like the ones created by PCA control, and they are not smooth, which is a major limitation since the motion appears non natural and hardly usable to perform some tasks (like carrying delicate objects). Moreover, the elbow starts to move with very small shoulder angular velocities, which does not make the prosthesis control very confortable nor robust;
- Control obtained through movement estimation by RBFN is less smooth than the one through estimation by PCA but it still remains within the natural baseline. The trajectory of these movements is close to natural movements (see *c*) and the predicted extensions are globally correct, except for the same targets as for LWR control. The delay between shoulder and elbow motions is close to the natural one.

RBFN seems thus to be the most suitable algorithm for elbow prosthetic movement-based control, among the three models considered in this study. Nonetheless, it cannot be concluded that RBFN really outperforms PCA and LWR and predicts a totally natural and accurate elbow motion. In particular, β_*final*_ is overpredicted for the highest targets, δ is still not close to zero (60 mm), and the body compensations are not smaller than with PCA and LWR control. Moreover, we can notice that each metric has an important standard deviation, be it for natural motions or the ones estimated by PCA, LWR or RBFN control. Indeed, beyond the common characteristics of reaching, each subject has its proper joint coordinations. This raises the following points : can we expect to find a model of joint coordinations that will perfectly perform for all subjects? To which extent the optimization of the regression algorithm used to build the interjoint coordination model can contribute in improving the control of the prosthesis? The results of this study show that, even if a global RBFN model (i.e., trained with data from several healthy subjects) has a good overall performance, the elbow extension is not correct enough to satisfy the accuracy required for the use of a prosthesis. Additional control schemes are needed.

The experiment performed to test the real-time response of the regression models also has some limitations, especially because the subjects wore the prosthesis as a supernumerary arm. Indeed, the artificial arm is not aligned with the shoulder, as in the case of amputated patients wearing a prosthesis, which can disturb the participant and might modify the natural shoulder/prosthetic elbow coordinations. The weight of the prosthesis and the weight repartition (different from the natural one, due to the motors and electronic parts) can destabilize the participants and partly explains the significant difference between “without-prosthesis” and “with-prosthesis” values of *a*. The real arm of the subjects (blocked in the orthosis) also hid the targets and reduced the visibility for some movements, especially when reaching the highest targets, which is one of the explanations of the bad predictions of elbow extension for these targets.

## 4. Conclusion and future works

This paper presents the experimental comparison of three regression models for movement-based control of a prosthetic elbow. This was performed through real-time tests, with human performing a reaching task with an arm prosthesis instead of their natural arm. Real-time tests are a significant contribution in movement-based control study since very few have been done so far (Bennett, 2016; Merad et al., 2016b). It is yet very important to take the prosthesis user in the loop as he reacts during the movement of the prosthesis and creates perturbations that cannot be studied in simulations or even in virtual reality environment. The three models were deliberately chosen among the simplest techniques in order to evaluate to which extent they can be performant, instead of immediately using more complex models (e.g., Multi-Layer Perceptron or multi-layer ANNs). We focused on reaching movements because, due to their high speed and the absence of concentration on intermediate joint control, they are absolutely adapted to movement-based control. Elbow flexion/extension was estimated from shoulder Euler angular velocities, computed in the trunk frame. The quantification of the prediction ability was assessed by six metrics (chosen as the most representative among sixteen), which accounted for task achievement, joint motion and body compensations. RBFN showed better performance than PCA and LWR. It predicted smooth enough movements, with a natural-like trajectory and correct timing but it does not reduce the body compensations nor always lead to a correct final elbow angle. An approximate interjoint coordinations modeling can also be done by PCA but it seems not performant enough to control a prosthesis, which requires very good predictions to satisfy the users. LWR predictions corresponded to the desired elbow extension angles but the problems of the smoothness of the output and the too sensitive response yet remain discriminating. Nevertheless, even if some performance differences exist between the models considered, none of them outperforms significantly the others. The regression technique used to model joint synergies may not be a key factor to improve prosthetic movement-based control.

This paper also highlights interesting elements to justify the use or the exclusion of some models for elbow/shoulder movement-based control. A sensible continuation of this study would first be to expand the comparison to more complex (multi-layer) ANNs, to evaluate if they are worthy or if the RBFN's ability is good enough to control a prosthesis. Moreover, the experiment conducted in this study remains perfectible. As said above, wearing the prosthesis as a supernumerary arm is not natural and raises some problems. Motions of healthy subjects and amputees are also different (Merad et al., 2018). It is known that upper limb amputees generally exhibit particular movement strategies and numerous body compensation strategies (for example, an overuse of the trunk; Metzger et al., 2012), because performing a task with a natural arm or with an artificial one remains a very different sensorimotor experience. The inter-subjects variability for amputees may also be higher than for non-amputees (different amputations, stump morphology, healing, etc.). Therefore, next experimental tests should be performed in a near future with final end users.

Finally, according to the results of this study that illustrate the rather little influence of the regression techniques and interjoint model on the control performance, we believe that new research directions should be explored. First, the individualization of the models could improve the prediction performance by tackling the issue of inter-subject variability. Future studies aim to directly build and improve the model on the user, taking into account his own coordinations, during first uses of the prosthesis with movement-based control. This is different from building the model with data from the remaining arm, which is a solution we do not consider as several studies have shown that joint coordinations of dominant and non-dominant arms are distinct (Bagesteiro, 2003; Sainburg et al., 2011; Schaffer and Sainburg, 2017). Second, “shared control paradigm” would offer an ability to the user to correct instantaneously the movement when the prediction was wrong or not adapted. This would also allow for voluntary control for smaller, more precise or slower movements.

## Author contributions

ML, MM, EdM, NJ, and AR-B conceived and designed the experiment. ML, MM, and EdM performed the participant registration and the experiment. ML, MM, and NJ analyzed the data. ML and NJ wrote the paper.

### Conflict of interest statement

The authors declare that the research was conducted in the absence of any commercial or financial relationships that could be construed as a potential conflict of interest.

## Appendix

### Complementary features for performance quantification of the regression models

**Task realization**

- Distance end-effector ratio
  characterizes the trajectory of the end-effector (the index finger). It is defined by $\frac{\text{total distance followed by the end-effector}}{\text{distance between initial and terminal points of the trajectory}}.$ It measures if the hand shakes during the movement or if there is bumps due to the regression. If it is the case, it provokes extra traveled distance. Distance end-effector ratio will also be higher if the elbow is too extended for the target;
- Smoothness of end-effector velocity
- Time to perform the task

**Articulation features**

- Smoothness of Euler angular velocities
  To know if the difference in smoothness for $\overset{\cdot }{\beta }$ comes from the models or from the inputs (possibility of sub-movements), we also looked at the smoothness of $\overset{\cdot }{\psi },\overset{\cdot }{\theta },\overset{\cdot }{\phi }$;
- Maximum of humerus aperture angle
  an important parameter to characterize and compare shoulder motions of healthy subjects and of subjects with the prosthesis;
- Elbow final altitude
  demonstrates if the participants did unnatural movements to compensate for bad $\overset{\cdot }{\beta }$ predictions by lifting their elbow;
- PCA distance between $\overset{\cdot }{\beta }$ and Euler angular velocities subspaces
  proposed by Bockemühl et al. (Bockemühl et al., 2010), measures the similarity of synergies between two movements.

**Body compensation**

- Amplitude (difference between end and beginning) of body inclination,
- Amplitude of body rotation,
- PCA distance between shoulder and trunk angles subspaces (Euler angles for shoulder and inclination/rotation angles for the trunk).
